# Role of Aliphatic Chain Characteristics on the Anti-Cracking Properties of Polymer-Modified Asphalt at Low Temperatures

**DOI:** 10.3390/polym11122025

**Published:** 2019-12-06

**Authors:** Peng Wang, Fu-quan Shi, Xi-yin Liu, Rui-bo Ren, Ying Zhu, Hui Sun, Guang-shun Zheng, Ze-jiao Dong, Li-zhi Wang

**Affiliations:** 1 School of Transportation Engineering, Shandong Jianzhu University, Jinan 250101, China; 15966651973@163.com (F.-q.S.); liuxiyin123@126.com (X.-y.L.); rrbgq@sdjzu.edu.cn (R.-b.R.); wlz85503@126.com (L.-z.W.); 2 Construction Management Branch of Qilu Transportation Development Group Co., Ltd., Jinan 250101, China; zhuying2005@126.com (Y.Z.); zhensunhui@126.com (H.S.); 18615663976@163.com (G.-s.Z.); 3 School of Transportation Science and Engineering, Harbin Institute of Technology, Harbin 150090, China

**Keywords:** modified asphalt, molecular structures characteristic, viscoelastic parameters at low temperatures, structure-performance correlation, burgers model

## Abstract

The anti-cracking properties of polymer-modified asphalt depend largely on the molecular structure of the polymer modifier. However, the mysterious structure-performance relationship is still elusive. In this paper, three kinds of polymers with different chain structures were selected to address this issue. The indices of styrene, trans-butadiene, aliphatic branched-chain, and aliphatic long-chain from the infrared spectrum were used to quantify the functional group compositions of polymer modifiers. Viscoelastic parameters, including relaxation time, dissipation energy ratios, and stiffness were assessed to illustrate the anti-cracking properties of polymer-modified asphalt. Results showed that relaxation time and dissipation energy ratios were mainly determined by the polymer network strength, molecular size, aliphatic chain feature, and the orientations speed of aliphatic chains. The short relaxation time and high dissipation ratio lead to the low stiffness and favorable low-temperature performance of asphalt. The improvement of these performances requires a polymer with high indices of an aliphatic long-chain, styrene, aliphatic branched-chain, and trans-butadiene, respectively. An aliphatic-long chain, aliphatic branched-chain, and trans-butadiene were soft segments in asphalt while styrene was the rigid segment. The soft segments affect the intramolecular friction, orientation, and thermal motion at low temperatures, whereas the rigid segment enhances the strength of polymer networks. Thus, the anti-cracking property of polymer-modified asphalt can be improved by adjusting the ratio of soft and rigid segments in the polymer modifier.

## 1. Introduction

Low-temperature cracking is the dominant mode of failure for asphalt pavement in cold climates. Asphalt materials serve as pavement binders. About 80% of the low temperature cracking resistance of asphalt pavement is caused by the poor performance of asphalt binders [1]. Pavement surface cracking is largely due to the low-temperature rheology performance of asphalt [2]. However, conventional straight asphalt (or conventional unmodified asphalt) exhibits poor resistance to low-temperature cracking and another distress, which can be addressed via asphalt modification [3].

Several types of modifiers can be used to improve the rheological properties of asphalt binders at low temperatures including polymers, acids, crumb rubber, etc. [4,5]. The most famous polymer modifier is the styrene-butadiene-styrene triblock copolymer (SBS) [6], which accounts for 80% of modified asphalts in China because of its excellent modification effect. The outstanding performance of SBS-modified asphalt is ascribed to its special molecular structures, that is, SBS can form a physical network, usually originates from the simultaneous presence of both rigid and flexible segments in its backbone [7]. The superior anti-cracking property of SBS-modified asphalt is due to its soft polybutadiene with branched-chain structures [3,8]. A common rule of free volume theory is that the flexibility of a polymer at low-temperature is closely related to the branched-chain structure in the polymer [9,10]. Weigel reported that the differences in the length and degree of branching in the molecules played an important role in the low-temperature performance of straight asphalt [11]. However, the relationship between the branching characteristics of polymers and the anti-cracking performance of modified asphalt is still elusive.

Moreover, the effective evaluation of the low-temperature performance of asphalt is the most important goal. A major contribution to the characterization of asphalt binders at low temperatures is the development of the performance grade (PG) system [12]. In PG, the bending beam rheometer (BBR) is a well-known test that is widely implemented for determining flexural-creep stiffness (*S*), creep compliance, and *m*-value of asphalt in low service temperature [13]. Based on BBR, Liu proposed the use of *m/S* and dissipated energy to reveal the physical relationship between low-temperature performance parameters and other parameters [14]. Some viscoelastic physical models, such as the Burgers model, and mathematical models, such as the Christensen-Anderson-Marasteanu model, are also applied to clearly elucidate the essential anti-cracking properties of asphalt [13,15,16,17]. However, the relationship between rheological properties at low-temperatures and the microstructure characters of polymer-modified asphalt is still unknown.

This paper aims to investigate the effect of the aliphatic chain structures of polymer modifiers on the anti-cracking properties of polymer modified asphalt. Three types of polymers with different molecular structures were selected. The length of branched-chain in polymers and modified-asphalt composite were determined by Fourier transformed infrared spectroscopy (FT-IR). A low-temperature condition was provided by a Bending Beam Rheometers (BBR). Burgers model and fractional viscoelastic model were used to obtain the viscoelastic parameters at low temperatures. Further, the viscoelastic parameters and molecular structure characteristic indexes were captured by the experiments and mathematical models. Finally, the relationship between viscoelastic parameters and the parameters of molecular structure characteristics were established. The work here will provide some new ideas to fully elucidate the anti-cracking mechanism of polymer-modified asphalt.

## 2. Experimental Materials and Design

### 2.1. Experimental Materials

**Figure 1:**
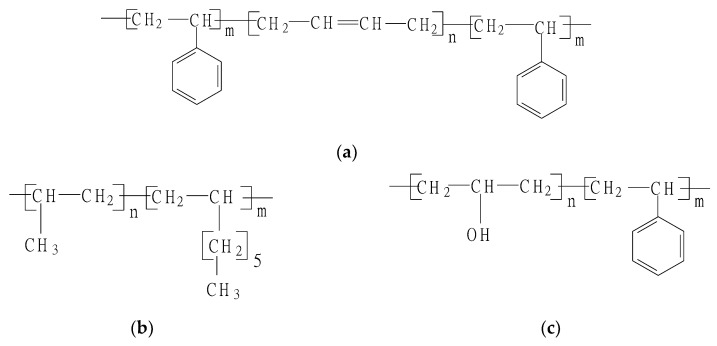
The Molecular structures of three polymer modifiers, (**a**) is the structure of styrene-butadiene-styrene block copolymers (marked as SBS), (**b**) is the structure of ethylene-octene copolymer (marked as POE), and (**c**) is the structure of styrene-propylene copolymers (marked as SPC).

Straight asphalt with penetration 70 dmm was used as based asphalt (maked as A70#). Three types of polymers were selected as asphalt modifiers, including liner type Styrene-Butadiene-Styrene block copolymers (marked as SBS, Baling branch of China petro-chemical corporation, Yueyang, China), ethylene-octene copolymer (marked as POE, LG Chemical Co., Ltd, Seoul, Korea) and styrene-propylene copolymers (marked as SPC, Baling branch of China petro-chemical corporation, Yueyang, China), as seen in Figure 1.

### 2.2. Preparation of Polymer-Modified Asphalt Samples

**Figure 2:**
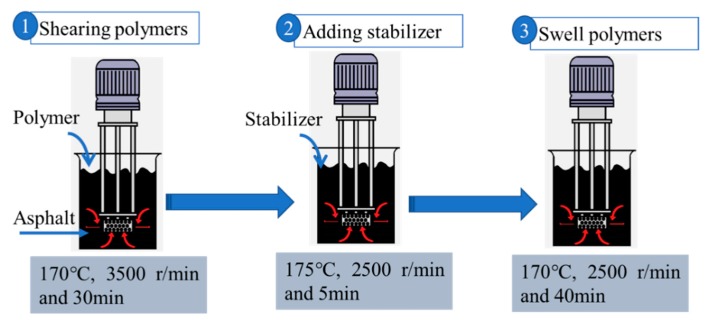
The preparation process of polymer modified asphalt.

Polymer-modified asphalt samples were prepared using a high-shear mixer (WeiYu Machine Co., Ltd., Shanghai, China). Base asphalt was heated at 160 °C for improved flow. SBS and POE or SPC were added to base asphalt at 170 °C for 30 min at a fixed rotation speed of 3000–3500 r/min. Thereafter, asphalt stabilizer (0.18 wt % by asphalt weight) was added into the mixtures at 175 °C for 5 min at a fixed rotation speed of 2500 r/min. Finally, the mixture temperature was decreased to allow the modifiers to swell polymers and prevent asphalt aging. Here, the asphalt stabilizer was a kind of sulfur crosslinking agent. The preparation process is listed in Figure 2. The amounts of SBS was 3 wt % by asphalt weight. The selection of SPC or POE amount was based on the attenuation of ductility, which satisfied the Chinese standard of SBS-modified asphalt I-D (ductility at 5 °C, ≥20cm). Adding POE within 2.5% or SPC 6.0% would satisfy this requirement. Thus, the contents of POE were 1.0%, 1.5%, and 2.0% of the total binder by weight, while the SPC amounts were 2.0%, 4.0%, and 6.0%.

### 2.3. Experimental Design

**Figure 3:**
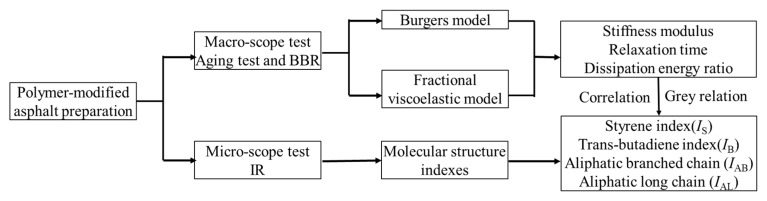
The flow of the whole experimental design.

The whole experimental design is summarized in Figure 3. In Figure 3, the low-temperature property of polymer modified asphalt was identified by BBR to obtain the stiffness (*S*) and *m*-value in accordance with ASTM D6648. The testing temperature is −12, −18 and −24 °C. The testing samples were the residue of Pressurized Aging Vessel. The structural characteristics of polymer modifiers and polymer modified asphalt are investigated using FT-IR. The sample for FT-IR was prepared by Infrared Window Daub Method on the Thermo Nicolet FT-IR instrument (Bruker, Karlsruhe, Germany), using KBr as the window material and scanning times 32.

## 3. Results and Discussions

### 3.1. Molecular Structure Characteristics of Polymer Modifiers

**Figure 4:**
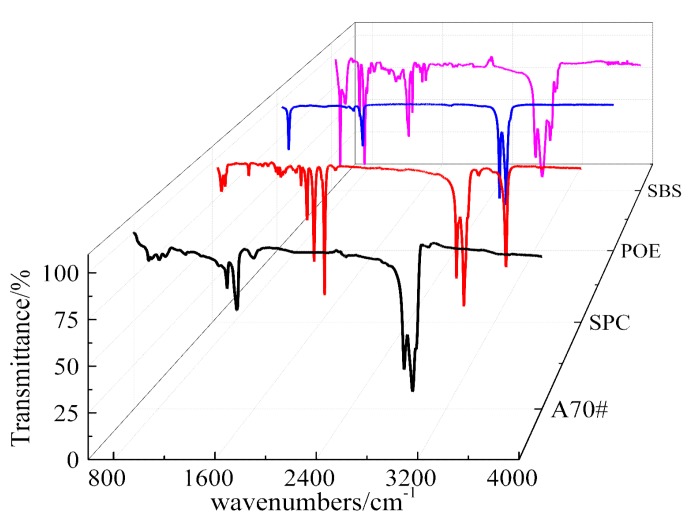
FT-IR spectrum of three polymer modifiers and base asphalt, the black curve is the spectrum of A70# (base asphalt), the red curve is the polymer of SPC, the blue one is the polymer of POE, and the purplish red one is the polymer of SBS.

Characteristic peaks in the FT-IR spectrum provided the functional group composition in the substance. Figure 4 summarizes the FT-IR spectrums of the three polymers. The peaks are divided into two sections in the FT-IR spectrum, namely functional group area (1000–4000 cm^−1^) and fingerprint area (400–1000 cm^−1^). In the functional group area, the observed wavenumbers at 2850, 2920, and 1450–1475 cm^−1^ demonstrated the existence of aliphatic long chain in saturated hydrocarbon. SBS showed a special functional group at a wavenumber at 3006 cm^−1^, which belonged to hydrocarbon stretching vibration in olefins. A wavenumber at 3310 cm^−1^ of SPC belonged to hydroxyl (OH^−^) [14,16,17,18]. In the fingerprint area, SBS showed characteristic peaks at wavenumbers of 699, 760, 910, and 966 cm^−1^, which belonged to benzene or aromatics. The peaks at 699 and 966 cm^−1^ belonged to the polystyrene phase of SBS [19,20,21,22]. The main functional group in POE was an aliphatic long-chain. The main functional groups in SPC were carbon-carbon double bond, aliphatic chain, benzene, and hydroxyl groups.

The functional group type and its relative content in polymers are listed in Table 1 based on the peak area ratio. Table 1 verified that the amount of aliphatic long-chain structures in the three polymers were different. The molecular structure characteristic parameters of the polymers are calculated by the peak areas, as shown in Equations (1)–(4).

(1) $$I_{s}=\frac{A_{699}}{A_{R}}\times 100\%$$

(2) $$I_{B}=\frac{A_{966}}{A_{R}}\times 100\%$$

(3) $$I_{AB}=\frac{A_{1377}{+A}_{1450-1475}+A_{2850}+A_{2920}}{A_{720}{+A}_{1377}{+A}_{1450-1475}+A_{2850}+A_{2920}}\times 100\%$$

(4) $$I_{AL}=\frac{A_{720}}{A_{720}{+A}_{1377}{+A}_{1450-1475}+A_{2850}+A_{2920}}\times 100\%$$

Here, *I*_S_ was styrene index, *I*_B_ was trans-butadiene index, the amount of aliphatic branched-chain was marked as *I*_AB_, and *I*_AL_ was used to perform aliphatic long chain in the polymer. The peak area at 699, 720, 966, 1377, 1450–1475, 2850 and 2920 cm^−1^ were marked as *A*_699_, *A*_720_, *A*_966_, *A*_1377_, *A*_1450–1475_, *A*_2850_, and *A*_2920_ respectively. *A*_R_ represents the total peak area between 500 to 4000 cm^−1^. Table 2 provided molecular structure characteristics parameters.

In Table 2, the aliphatic long-chain index of SBS was the smallest, POE was the largest, and SPC was in the middle. Thus, SBS-modified asphalt was selected as the controller, and SBS-modified asphalt with different POE or SPC amounts were used in subsequent research to investigate the effect of aliphatic long-chain structure on the anti-cracking ability of polymer-modified asphalt at low temperature.

### 3.2. Relaxation Time at Low Temperature Based on the Burgers Model

As a viscoelastic material, asphalt exhibits changing strain with time under constant stress conditions, but the viscoelastic behaviors of this process can be described based on the physical mechanics model. The commonly used physical mechanics model to express creep deformation at low temperature is the four-parameter Burgers model, which is composed of two spring elements and two sticky pot elements in series and parallel [23]. Thus, the four-parameter Burgers model was used to calculate the viscoelastic parameters at low temperatures in this paper. The constitutive relation of the four-parameter Burgers model is expressed in Equation (5), as follows:

(5) $$\frac{1}{S(t)}=\frac{\varepsilon \left(t\right)}{\sigma_{0}}=\frac{1}{E_{1}}+\frac{t}{\eta_{1}}+\frac{1}{E_{2}}\left(1-\exp\left(-\frac{E_{2}}{\eta_{2}}t\right)\right)$$

where *S*(*t*) is the stiffness modulus, *E*_1_ is the instantaneous modulus of elasticity, and *η*_1_ is the viscosity coefficient. *E*_2_ and *η*_2_ are viscous indexes of asphalt binders when the deformation could not disappear immediately under load revocation and load slowly deformed under loading condition. *σ*_0_ is the constant stress amplitude. *E*_1_, *η*_1_, *E*_2_, and *η*_2_ are model parameters, obtained using the solution for nonlinear programming, as seen in Table 3.

As presented in Table 3, *η*_1_ was considerably larger than *η*_2_, whereas *E*_2_ was slightly higher than *E*_1_ at each temperature point for one asphalt sample. With decreasing temperature, all four parameters gradually increased. At the same condition, POE largely affected on *E*_1_, *η*_1_, and *E*_2_, whereas SPC largely affected *η*_2_.

**Figure 5:**
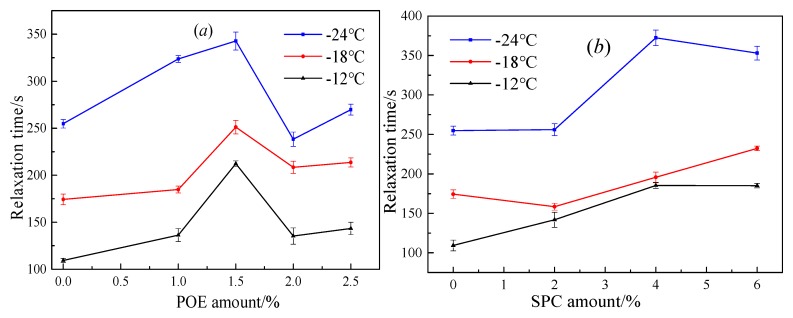
Effect of polymers amount on the relaxation time for SBS-modified asphalt, (**a**) is the effect of POE amount on the relaxation time, and (**b**) is the effect of SPC amount on the relaxation time.

For viscoelastic materials, relaxation time is the ratio of *η*_1_/*E*_1_, as listed in Figure 5. For SBS-modified asphalt with POE, the relaxation time considerably increased and decreased when the additive of POE was <1.5% and >2.0%, respectively. Relaxation time refers to the time required for a material to transition from an equilibrium state to a new equilibrium state under external force loading conditions. Thus, adding POE at >2.0% enhances the relaxation ability of SBS-modified asphalt, thereby finally slowing down the stress concentration in modified asphalt under external force loading conditions. A peak value of the POE amount was observed during the relaxation time. However, the peak value changed with temperature and POE amount.

For SBS-modified asphalt with SPC, the relaxation time of composited modified asphalt was increased with SPC amount. Thus, additional time was needed to reach a new equilibrium under the same force loading conditions. Consequently, a short relaxation time provided an improved anti-cracking property of asphalt. Table 2 shows a comparison of the molecular structure characteristics of POE and SPC. The aliphatic long-chain index of POE was larger than that of SPC, but SPC holds larger aliphatic branched-chain index than that of POE. Here, the aliphatic long chain was an aliphatic structure with a carbon number of not less than four. Therefore, the increasing aliphatic long-chain and decreasing aliphatic branched-chain in the polymer modifier provided an improved low-temperature performance of asphalt. The relaxation time depended on the molecular size and orientation speed of different molecules. The aliphatic long-chain was easier to orient due to its regular structure and small molecular size. Although the aliphatic branched-chain possessed a smaller molecular size than the aliphatic long chain, the thermal motion was sufficiently intense to prevent quick orientation and reach a new equilibrium. Thus, aliphatic long-chain positively affects the decrease of asphalt binder relaxation time.

### 3.3. Dissipation Energy Ratio Based on Fractional Viscoelastic Model

Fractional viscoelastic model is a powerful tool to recognize the viscous-elastic behavior of polymer within a broad frequency and time range, which is expressed the rule of creep compliance, as shown in Equation (6) [24].

(6) $$S\left(t\right)=\frac{1}{J\left(t\right)}=\frac{1}{A}t^{-a}$$

(7) lg(S(t))=−lgA−αlg(t)

(8) $$m\left(t\right)=\left|\frac{d\left\{-\mathrm{lg}A-\alpha \mathrm{lg}\left(t\right)\right\}}{d\left\{\mathrm{lg}\left(t\right)\right\}}\right|=\alpha$$

where *S*(*t*) is creep stiffness, *J*(*t*) is creep compliance, *t* is loading time, and *A* and *α* are the constants of model parameters. *S*(*t*) at different temperatures can be captured from the BBR test. *m*(*t*) is the tangent slop on the logarithmic curve of creep stiffness and logarithmic curve of time at 60 s. Thus, the equation can be deduced to Equation (8). Table 4 provides the viscoelastic parameters of the fractional viscoelastic model.

The results in Table 4 showed that adding POE or SCP slightly decreased *α* for SBS-modified asphalt. A higher *A*-value was observed for a sample with SCP but not with POE. Most values of *A* and *α* were all decreased with POE or SCP amount. However, *A* and *α* could not be used to explain the molecular movement during loading. Thus, the dissipation energy ratio (ϖ) was proposed.

Dissipation energy is commonly used to express the energy dissipated by the viscous flow of materials due to temperature variations [25,26]. This part of the energy is not recoverable. Thus, ϖ was used to evaluate the low-temperature property of viscoelastic materials.

(9) $$\varpi =\frac{W_{d}\left(t\right)}{W_{s}\left(t\right)}=\frac{2^{\alpha -1}}{1-2^{\alpha -1}}$$

(10) $$W_{d}\left(t\right)=\frac{1}{2}\sigma_{0}^{2}A{\left(2t\right)}^{\alpha }$$

(11) $$W_{s}\left(t\right)=A\sigma_{0}^{2}\left(t^{\alpha }-\frac{1}{2}{\left(2t\right)}^{\alpha }\right)$$

*W_d_*(*t*) is the loss energy, and *W_s_*(*t*) is the storage energy; these are obtained from Equations (10) and (11) by using the fractional viscoelastic model. *σ*_0_ is the loading stress, *t* is loading time, and *α* and *A* are constant parameters of the fractional viscoelastic model.

**Figure 6:**
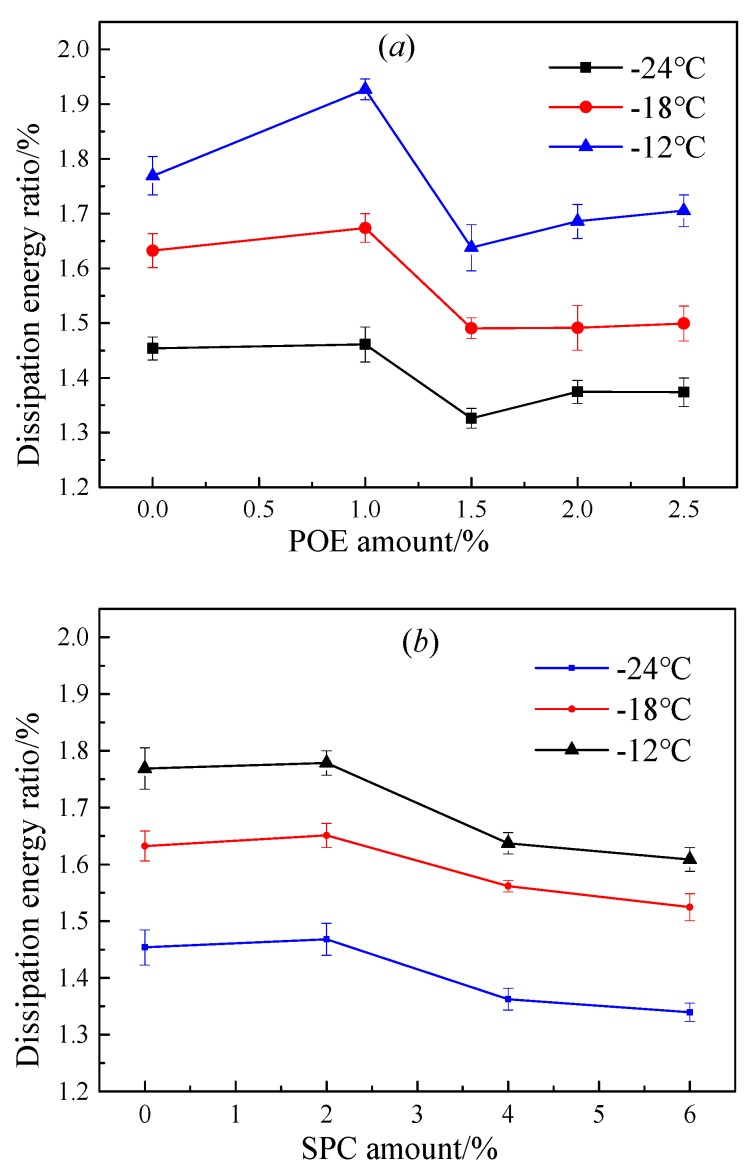
Effect of polymers amount on the dissipation energy ratio for SBS-modified asphalt, (**a**) is the effect of POE amount on the dissipation energy ratio, and (**b**) is the effect of SPC amount on dissipation energy ratio.

Figure 6 demonstrates that ϖ decreases with temperature. For the asphalt sample with POE, ϖ at −12 °C initially increased and then decreased with increasing POE amount, whereas ϖ at −18 °C and −24 °C decreased with the POE amount. For the asphalt sample with SPC, the values of ϖ were reduced sharply when the SPC amount was >2.0%. The dissipation energy ratio was used to express the ability to overcoming internal friction among molecules, which closely related to temperature and microstructure features. For temperature, the higher the temperature, the more intense the Brownian motion is. Consequently, the dissipated energy of the asphalt sample was larger at high temperatures. For microstructure features, two factors affect the dissipated energy ratio, including the features of a polymer network in asphalt and the molecular structure of the polymer. A dense polymer network would decrease free volume and increase internal friction. The large steric hindrance and many-branched chains also consumed much energy during movement. Thus, adding a polymer with additional aliphatic chains would enhance its dissipation energy ratio. However, adding POE at <1.5% or SPC at <2.0% into SBS-modified asphalt, decreased the dissipation energy ratio. The excessive polymer with an aliphatic chain may negatively effect the distribution of the polymer network.

Only the sample with 1.0% POE at −12 °C showed a higher dissipation energy ratio than that of pure SBS-modified asphalt, whereas the sample with an SPC of <2.0% showed higher dissipation energy ratio than that of pure SBS-modified asphalt. SBS-modified asphalt with the same POE showed lower dissipation energy ratio than that with SPC because the network of SPC and SBS were slightly denser than that with POE due to the lack of rigid segment. A large dissipation energy ratio would improve the low-performance of asphalt. Therefore, adding SPC at <2.0% would improve the anti-cracking property of SBS-modified asphalt better than adding POE.

### 3.4. Relationship between Molecular Structure and Viscoelastic Parameters

**Figure 7:**
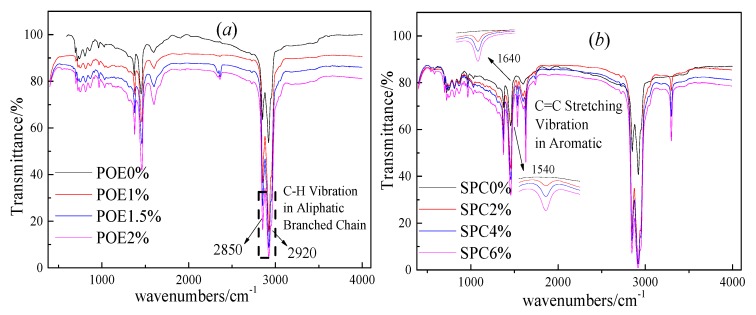
Effect of polymers amount on FT-IR spectrum of SBS-modified asphalt, (**a**) is the effect of POE amount on FT-IR spectrum, and (**b**) is the effect of SPC amount on FT-IR spectrum.

The FT-IR spectrum of SBS modified asphalt with different POE or SPC amounts were obtained from Infrared Window Daub method, as shown in Figure 7. For SBS-modified asphalt with POE, the wavenumber at 2850 and 2920 cm^−1^ belonged to POE in Figure 7a. For the SBS-modified asphalt with SPC, the intensities of the characteristic peak at 1540, 1640, and 3310 cm^−1^ wavenumbers were remarkably increased with SPC amount, as shown in Figure 7b. In contrast with those shown in Figure 4 and Figure 7, the characteristic peaks of composite-polymer modified asphalt were composed of polymer and base asphalt, and no new peaks appeared after modification. Thus, no chemical reaction occurred among SPC or POE, SBS, and base asphalt.

**Figure 8:**
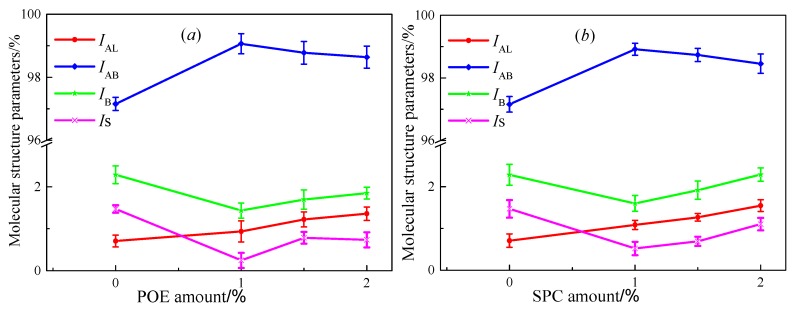
Effect of polymers amount on molecular structure characteristics parameters of SBS-modified asphalt, (**a**) is the effect of POE amount on molecular structure characteristics parameters, and (**b**) is the effect of SPC amount on molecular structure characteristics parameters.

The characteristic peak indexes were calculated using Equations (1) and (2), as shown in Figure 8. The SBS-modified asphalt showed the largest values of styrene index and trans-butadiene index, whereas those indexes showed almost no change with POE or SPC amount due to the absence of polystyrene and butadiene in POE and SPC. The aliphatic long-chain indexes, saturated aliphatic hydrocarbon indexes, and aliphatic branched-chain increased with POE or SPC amount due to the aliphatic chains. Those branched and long chains in polymer greatly affected the macro anti-cracking properties of composite-polymer modified asphalt. Thus, the relationship between anti-cracking property and the branched chains was determined using a grey correlation.

**Figure 9:**
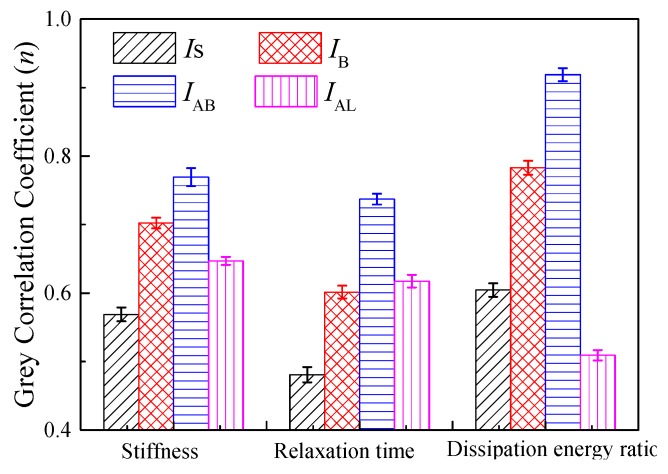
Grey correlation coefficient between molecular structure characteristics parameters and rheological properties based on grey correlation method.

Figure 9 provides the grey correlation coefficient (*n*) between molecular structure indexes and viscoelastic parameters from the Burgers model and fractional viscoelastic model. A large *n*-value implied a high correlation between two parameters. Stiffness was mainly dependent on the aliphatic-branched chain and trans-butadiene index, indicating that a high stiffness was obtained when the amount of aliphatic-branched chain and trans-butadiene was large in the polymer modifier. The smaller the stiffness modulus was, the stronger the deformation ability of asphalt at low-temperature hold. Therefore, the result in Figure 8 demonstrated that a polymer with a high aliphatic long-branched chain and styrene index would provide a small stiffness and improved anti-cracking performance for asphalt.

For relaxation time, the largest grey correlation coefficient was the aliphatic-branched chain index, the trans-butadiene index was slightly lower than the aliphatic long-branched chain index, and the smallest was the styrene index. Thus, the results in Figure 9 implied that a polymer with high styrene, trans-butadiene, and aliphatic long-chain amount but the small aliphatic branched-chain could exhibit the good anti-cracking property of asphalt.

Short relaxation time and large dissipation ratio commonly cause small stiffness and good low-temperature performance of asphalt. Small stiffness requires a polymer with a high aliphatic long chain and large styrene. A polymer with a large aliphatic branched-chain requires a high dissipation energy ratio, as shown in Figure 9. The low-temperature performance of asphalt binder mainly depended on the deformation capacity and toughness at low temperatures. The deformation capacity relied mostly on the molecular size ascribed to the steric hindrance effect [27]. Trans-butadiene and aliphatic showed a smaller steric hindrance than styrene. Thus, styrene was a rigid segment, whereas the other groups were soft segments in the polymer. The high proportion of soft segment in polymer performed a good deformation capacity of asphalt binders. The toughness of the modified asphalt was mainly dependent on the strength of the polymer network. A polymer with a soft segment and a rigid segment shows combined hardness and softness, providing a large network strength if it exhibits good compatibility with asphalt. A large network strength increases the toughness of asphalt. A polymer with high trans-butadiene and large styrene requires a short relaxation time. Clearly, trans-butadiene was easier to move because of small molecular size at a given temperature. Thus, enhancing the content of trans-butadiene could increase the deformation capacity of asphalt. The aliphatic branched-chain showed increased intramolecular friction due to its irregular structure, finally dissipating additional energy at a given temperature. Therefore, the dissipation energy ratio was mainly dependent on the aliphatic branched-chain. Aliphatic branched-chain exhibited difficulty in orientation, resulting in large relaxation time and large stiffness.

## 4. Conclusions

The unclear correlation between the molecular structure and rheological performance of asphalt material has attracted wide attention for over half a century of intensive research. However, what molecular structure can improve the properties of asphalt is still unclear. Thus, three kinds of polymers with different chain structures were selected. This paper aimed to reveal the relationship between aliphatic chain characteristics and the anti-cracking properties of polymer-modified asphalt.

The functional group compositions of polymer modifiers were investigated from the infrared spectrum. Styrene index, trans-butadiene index, aliphatic branched-chain index, and aliphatic long-chain index were used to quantify the molecular structure characteristics of polymers by using an infrared spectrum. POE was a polymer with a large aliphatic long-chain index, whereas SPC was a polymer with a large aliphatic branched-chain index. SBS was a polymer with a trans-butadiene and styrene segment.

Relaxation time based on the Burgers model and dissipation energy ratio based on the fractional viscoelastic model were calculated using the BBR data. The results showed that the relaxation time of SBS-modified asphalt was decreased when POE at >2.0% was added, whereas the addition of SPC increased the relaxation time. The dissipation energy ratios initially increased and then decreased as the POE amount increased but decreased sharply when the SPC added was >2.0%. SPC performed a greater ability to increase the dissipation energy ratio than POE. These results were ascribed to the strength of the polymer network, molecular size, aliphatic chain feature, and the orientation speed of aliphatic chains.

The relationship between the molecular structure and macro viscoelastic parameters was captured by the grey correlation coefficient. The aliphatic branched-chain increased intramolecular friction but showed difficulty in orientation due to the irregular structure, thereby resulting in a high dissipation energy ratio, relaxation time, and stiffness. The aliphatic long-chain decreased the stiffness and relaxation time except for the dissipation energy ratio. In summary, small stiffness required by a polymer with a high aliphatic long chain and large styrene. A high dissipation energy ratio is required by a polymer with a large aliphatic-branched chain. Short relaxation time is required by a polymer with high trans-butadiene and large styrene. Thus, an improved anti-cracking property of polymer-modified asphalt can be obtained if the polymer exhibits a suitable ratio of soft segment and a rigid segment.

## Figures and Tables

**Table 1:** Functional group type and its relative content of polymer modifiers and base asphalt.

Wavenumbers/cm^−1^	Functional Group	Chemical Type	Functional Group Amount/%			
			A70#	SBS	POE	SPC
699	Stretching vibration of C=C in benzene	Polystyrene	0	8	0	0
720	Methylene(–CH_3_) chain synergy vibration (*n* ≥ 4)	Aliphatic long chain (saturate)	4	0	10	1
760	Aromatic branched chain bending vibration	Aromatic	4	7	0	0
910	Benzene stretching vibration	Aromatic	0	3	0	0
966	Butadiene stretching vibration	Butadiene	0	8	0	0
1377	Methyl (–CH_3_) umbrella vibration	Aliphatic branched chain (saturate)	13	0	1	1
1450–1475	Methylene (–CH_3_) shear type vibration	Aliphatic branched chain (saturate)	16	9	10	4
1493	Asymmetric benzene ring breathing vibration	Benzene ring and carboxyl	0	2	0	0
1540	Carbon-carbon double bond (C=C) stretching vibration	Aromatic	0	0	0	9
1640	Carbon-carbon double bond (C=C) stretching vibration	Aromatic	0	0	0	10
2850	Methyl (–CH) symmetry vibration	Aliphatic branched chain (saturate)	20	24	29	29
2920	Methyl (–CH) symmetry vibration	Aliphatic branched chain (saturate)	42	29	49	29
3006	Hydrocarbon stretching vibration in olefins	Olefins	0	10	0	0
3310	Hydroxyl (OH^−^) stretching vibration	Hydroxyl	0	0	0	17

**Table 2:** Molecular structure characteristics parameters of polymer modifiers.

Polymer Type	*I*_AL_/%	*I*_AB_/%	*I*_B_/%	*I*_S_/%
SBS	0.00	100.00	10.29	9.70
POE	9.69	90.31	0.00	0.00
SPC	2.07	97.93	0.00	0.00

**Table 3:** Viscoelasticity parameters at low temperature using burgers model.

Polymer Amount	Temperature/℃	*S*/MPa	*E*_1_/MPa	*η*_1_/MPa·s	*E*_2_/MPa	*η*_2_/MPa·s
SBS3.0%	−12	109	271	29,620	235	8013
	−18	194	403	70,242	409	17,344
	−24	503	917	233,650	1198	45,323
SBS3.0% + POE1%	−12	136	274	30,500	166	28,490
	−18	332	638	117,886	749	37,051
	−24	668	1067	345,387	2131	73,917
SBS3.0% + POE1.5%	−12	170	283	73,152	330	30,512
	−18	322	578	145,182	772	32,887
	−24	704	1098	376,361	2423	73,965
SBS3.0% + POE2%	−12	176	422	57,107	349	12,463
	−18	383	739	154,028	945	30,511
	−24	589	1105	263,314	1719	28,297
SBS3.0% + POE2.5%	−12	105	235	33,689	213	8756
	−18	366	718	153,394	838	28,452
	−24	618	1043	281,342	2029	51,642
SBS3.0% + SCP2%	−12	93.5	227	32,202	168	6982
	−18	174	392	62,099	340	13,032
	−24	446	811	207,665	1203	32,917
SBS3.0% + SCP4%	−12	71.5	150	27,796	143	6398
	−18	171	353	69,116	359	13,432
	−24	560	950	353,715	1387	49,496
SBS3.0% + SCP6%	−12	95	207	38,295	184	7326
	−18	179	345	80,141	387	15,640
	−24	523	880	310,666	1485	44,056

**Table 4:** The viscoelastic parameters of composite-polymer modified asphalt using fractional viscoelastic model.

Polymer Type and Amount	−12 °C		−18 °C		−24 °C	
	*A*/Pa	*α*	*A*/Pa	*α*	*A*/Pa	*α*
SBS3.0%	0.00222	0.3535	0.00148	0.3106	0.00075	0.2448
SBS3.0% + POE1%	0.00157	0.3969	0.00092	0.2974	0.00067	0.2008
SBS3.0% + POE1.5%	0.00168	0.3124	0.00110	0.2594	0.00067	0.1893
SBS3.0% + POE2%	0.00152	0.3281	0.00092	0.2597	0.00073	0.2113
SBS3.0% + POE2.5%	0.00249	0.3342	0.00095	0.2628	0.00070	0.2110
SBS3.0% + SCP2%	0.00265	0.3445	0.00161	0.3168	0.00091	0.2258
SBS3.0% + SCP4%	0.00395	0.3122	0.00185	0.286	0.00078	0.2060
SBS3.0% + SCP6%	0.00310	0.3025	0.00187	0.2724	0.00087	0.1955
